# 2901. Associations between climate and diabetic foot infection microbiology: a systematic review and meta-analysis

**DOI:** 10.1093/ofid/ofad500.172

**Published:** 2023-11-27

**Authors:** Benjamin Rabin, Sophie M Lockwood, Ellen Martinson, Kyra Urquhart-Foster, Priyanka Bhanushali, Julia Raymond, Meg McAloon, Jillian Dunbar, Baffour Otchere, Mia S White, Marcos Schechter

**Affiliations:** Brigham & Women's Hospital, Atlanta, GA; Emory University Rollins School of Public Health, Atlanta, Georgia; Emory University Rollins School of Public Health, Atlanta, Georgia; Medical College of Georgia at Augusta University, Augusta, Georgia; Emory University Rollins School of Public Health, Atlanta, Georgia; Emory University Rollins School of Public Health, Atlanta, Georgia; Emory University Rollins School of Public Health, Atlanta, Georgia; Emory University Rollins School of Public Health, Atlanta, Georgia; Emory University, Atlanta, Georgia; Emory University, Atlanta, Georgia; Emory University School of Medicine

## Abstract

**Background:**

Diabetic foot infections (DFIs) are the leading cause of preventable limb loss globally. The International Working Group on Diabetic Foot Infection guidelines advise an empiric antibiotic regimen choice for DFI partly based on climate: gram-negative and *P. aeruginosa* active antibiotics are recommended for people residing in subtropical and tropical climates with moderate and severe DFIs. However, there is limited data supporting this recommendation. Our systematic review and meta-analysis aimed to understand the prevalence of gram-negative and *P. aeruginosa* DFIs across solar climate zones (temperate, subtropical, and tropical).

**Methods:**

We searched PubMed, Embase, AIM, IMSEAR, IMEMR, LILACS, and WPRIM for studies published in any language between 2010-2020 reporting ≥5 unique patients with a DFI (Figure 1). Two reviewers independently assessed studies for eligibility, and we used double data entry. The senior author resolved disagreements. We assigned climate zone based on study center location and built Forest plots to depict the proportion of patients with (1) ≥1 gram-negative bacteria and (2) with *P. aeruginosa*­ in foot soft tissue and/or bone cultures. We reported common and random effects models.

**Figure:**
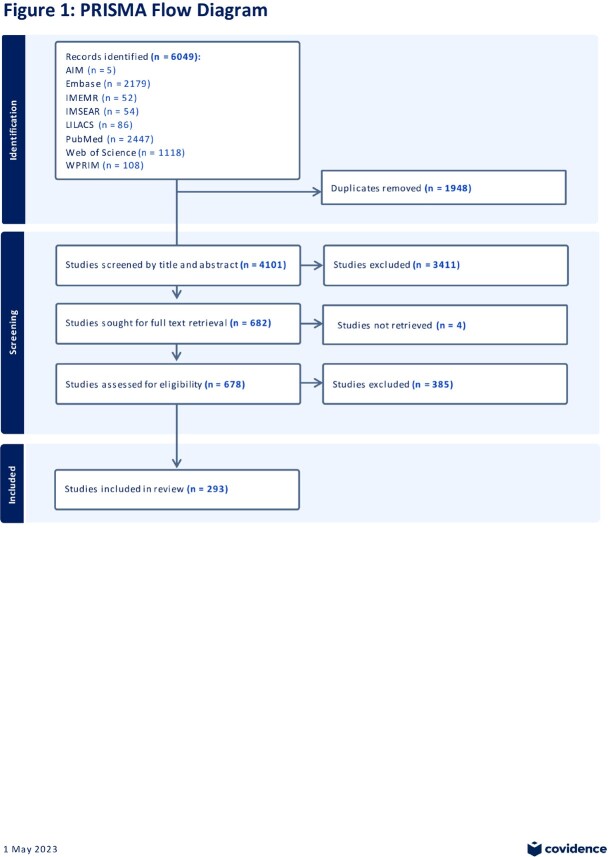

**Results:**

293 studies out of 4101 studies met our inclusion criteria. Of these, 53 studies representing 4,970 patients reported the proportion of patients with gram negative infections, and 270 studies representing 36,026 patients reported the proportion with *P. aeruginosa*. A random effects model found the proportion of patients with gram negative infection varied by climate zone: 40% (95% confidence interval 33% – 54%) in temperate regions; 58% (95% CI 51% – 65%) in sub-tropical regions; and 67% (95% CI 49% – 61%) in tropical regions (Figure 2). The proportion of DFI with *P. aeruginosa* was 14% (95% CI 12% - 17%) in temperate areas; 15% (95% CI 13% - 17%) in subtropical regions; and 16% (95% CI 14 – 17%) in tropical regions (Figure 3).

**Figure 2:**
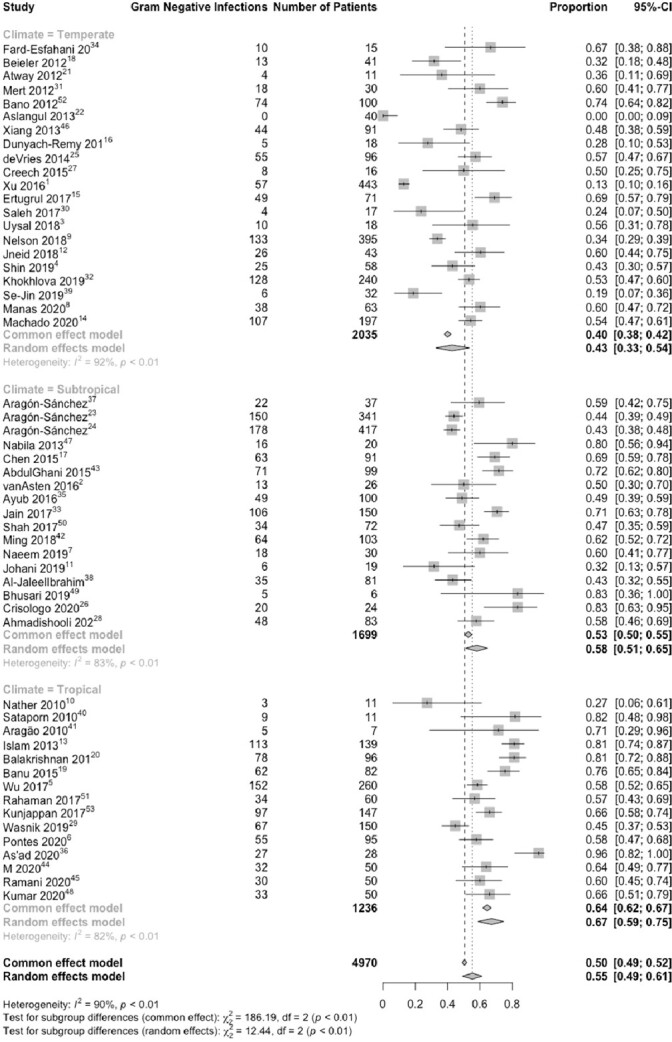
Meta analysis of the proportion of gram negative DFI infection by solar climate

**Figure 3:**
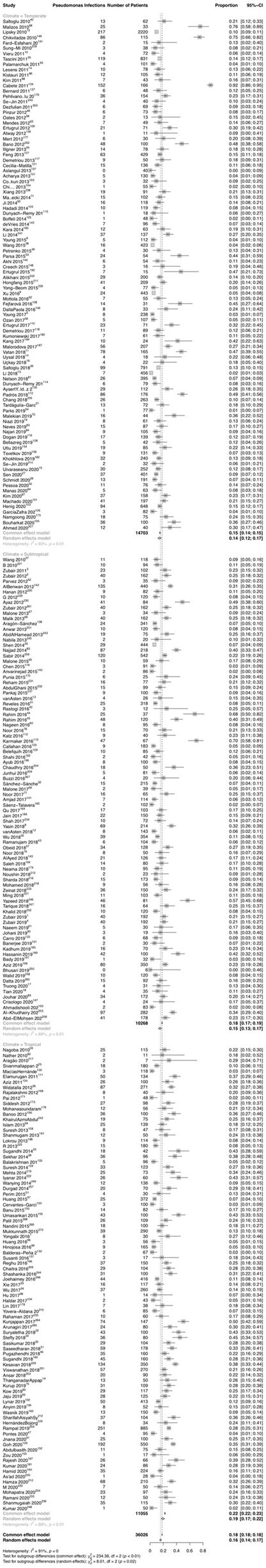
Meta analysis of the proportion of Pseudomonas aeruginosa DFI infection by solar climate

**Conclusion:**

Our meta-analysis suggests that gram-negative infections occur at higher proportions in tropical and sub-tropical areas compared to temperate regions. Future studies should aim to elucidate the interactions between other risk factors for gram-negative and/or *P. aeruginosa* DFIs (e.g., antibiotic exposure, infection severity) and climate.

**Disclosures:**

**All Authors**: No reported disclosures

